# Lineage‐specific genomics: Frequent birth and death in the human genome: The human genome contains many lineage‐specific elements created by both sequence and functional turnover

**DOI:** 10.1002/bies.201500192

**Published:** 2016-05-27

**Authors:** Robert S. Young

**Affiliations:** ^1^ MRC Human Genetics Unit, MRC IGMMUniversity of EdinburghEdinburghUK

**Keywords:** enhancers, evolution, indels, lineage‐specific biology, promoters, transcriptional regulation

## Abstract

Frequent evolutionary birth and death events have created a large quantity of biologically important, lineage‐specific DNA within mammalian genomes. The birth and death of DNA sequences is so frequent that the total number of these insertions and deletions in the human population remains unknown, although there are differences between these groups, e.g. transposable elements contribute predominantly to sequence insertion. Functional turnover – where the activity of a locus is specific to one lineage, but the underlying DNA remains conserved – can also drive birth and death. However, this does not appear to be a major driver of divergent transcriptional regulation. Both sequence and functional turnover have contributed to the birth and death of thousands of functional promoters in the human and mouse genomes. These findings reveal the pervasive nature of evolutionary birth and death and suggest that lineage‐specific regions may play an important but previously underappreciated role in human biology and disease.

**Abbreviations**
CAGE: cap analysis of gene expression
ChIP: chromatin immunoprecipitation
DHS: DNaseI hypersensitive site
H3K27ac: acetylated histone H3 lysine 27
H3K4me2: dimethylated histone H3 lysine 4
H3K4me3: trimethylated histone H3 lysine 4
lncRNA: long non‐coding RNA
LTR: long terminal repeat
miRNA: microRNA
TF: transcription factor

## Introduction

The large and varied diversity observed between individuals and across species is reflected in high levels of genetic diversity. The study of this diversity between mammalian species has been possible on a genome‐wide scale since the publication of the first complete drafts of the human and mouse genomes in 2001 1 and 2002 2, respectively. The subsequent emergence of next‐generation sequencing technologies 3 has led to an explosion of whole‐genome sequencing, such that public databases now host many mammalian genomes, and 39 of these can currently be directed viewed and compared through the Ensembl genome browser (www.ensembl.org) 4. The first personal genome sequence was only published in 2007 5, but has now been joined by a number of studies, including the 1,000 Genomes Project which sequenced over 1,000 individual genomes 6 and those from deCODE Genetics which sequenced over 2,500 individual genomes from the Icelandic population 7.

This wealth of data has stimulated the field of comparative genomics, which investigates both the similarities and differences between genomes. Much early work focussed on identifying shared features between sequenced genomes and restricted itself to the small proportion of the genome which encodes for protein‐coding genes. Many of these genes have been deeply conserved throughout evolution from yeast to human, the lineages of which diverged approximately one billion years ago 8. The number of protein‐coding genes found in vertebrate species is relatively constant and, unexpectedly, does not appear to correlate with our assumptions regarding organismal complexity 9.

Not all protein‐coding genes, however, are evolutionarily ancient. For example, *C20orf203* is found only in the human genome, and is absent from closely related primates. This gene is highly expressed in the brain, and is further upregulated in Alzheimer's disease, which suggests a potential role for this lineage‐specific gene in the development of the disease 10. There are now several reports of genes that have been born and died in various species, through a variety of mechanisms (fully reviewed by Kaessmann 11).

Comparative genomics has also been applied to studying the remaining, non‐coding, regions of the genome, which make up almost 99% of the genome 12 and contain a wealth of transcriptional and regulatory elements. MicroRNAs (miRNAs) – short, approximately 22 nt long, non‐coding RNA genes primarily involved in negative regulation of protein‐coding genes 13 – are often deeply conserved across a range of divergent species 14. Long non‐coding RNAs (lncRNAs) are a relatively unstudied class of non‐coding transcripts that are over 200 bp long 15. lncRNAs show modest evolutionary constraint, which has been interpreted as indicating that these sequences have been conserved across species because they encode a biological function 16, 17, 18. Similarly, non‐coding regulatory elements such as enhancers, which positively regulate gene expression at a distance 19, have been computationally predicted in regions which show increased evolutionary conservation across species. This approach has been demonstrated to have a 45% success rate in predicting enhancers using comparative genomics alone 20.

As for protein‐coding genes, there are also corresponding examples of non‐coding RNA genes that have been emerged during evolution, such as the mouse‐specific lncRNA *Poldi*. This lncRNA is restricted to the post‐meiotic cells of the testis, and promotes sperm motility and testis development 21. Similarly, the miRNA *miR‐941* – which was born in the human lineage between one and six million years ago from the expansion of an evolutionarily unstable tandem repeat sequence – was recently discovered to be important for neurotransmitter signalling in the brain 22.

There are a handful of known examples of enhancer birth and death in the human genome. For example, the non‐coding element *HACNS1* has evolved rapidly in humans and only the human sequence, but not the orthologous primate sequence, is able to function as a limb enhancer in mouse reporter experiments 23. Alternatively, an enhancer for the *AR* gene, which is conserved across most mammalian species has been deleted from the human genome. The activity of this enhancer is correlated with the formation of whiskers and penile spines in non‐human mammals, and it has been speculated that this loss in humans may be linked to increased monogamous reproductive strategies relative to other primates 24.

On a genome‐wide scale, non‐coding elements are less conserved between mammalian species than protein‐coding genes 25, 26. This has led many to speculate that organismal diversity is not in fact driven by changes to the protein‐coding gene set, but by divergence in the regulatory mechanisms responsible for controlling their expression 27. Due to their increased volatility, much recent work has therefore focussed on the birth and death of such non‐coding, regulatory elements within the human genome.

In this essay, I will first discuss sequence turnover, where sequence is either inserted (born) or deleted (dies) along a lineage. I will then describe analyses of functional elements that have been identified through experimental profiling (Box 1) and were subsequently shown to completely turn over between lineages despite conservation of the underlying DNA sequence (functional turnover). Where such profiling has been done in multiple species, it is possible to further define functional turnovers as gains and losses down individual lineages and throughout this essay I will also refer to these as birth and death events, respectively. Finally, I will examine those studies that have considered both the birth and death of sequence and function in the same experimental system. I will show that the birth and death of entire regulatory elements are frequent occurrences within the human genome, and will suggest that future research is likely to focus on both the transcriptional regulatory and phenotypic consequences of these events to normal and perhaps also pathogenic human diversity.

Box 1 **Functional genomics technologies discussed in this review**

1. Chromatin immunoprecipitation followed by sequencing (ChIP‐seq): Identifies the locations of histone modifications or the sites of a DNA‐binding protein, such as a transcription factor, by high‐throughput sequencing of DNA pulled down with an antibody specific to the modification or protein of interest.
2. DNase 1 hypersensitivity sites sequencing (DNase‐seq): Discovers all classes of active regulatory elements by digesting accessible chromatin which is not packaged into nucleosomes, followed by high‐throughput DNA sequencing.
3. Cap analysis of gene expression (CAGE): Clones the 5′ ends of transcribed mRNA molecules and then subjects them to high‐throughput sequencing to precisely identify the sites of transcription initiation at promoters and other transcribed elements, such as enhancers.

## Sequence turnover is common in the human genome

**Figure 1:**
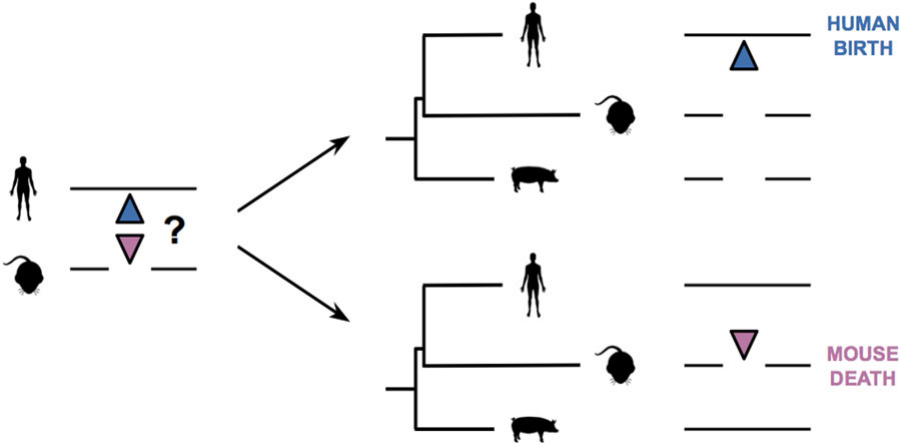
By comparison to a third outgroup species, the lineage along which a mutation took place can be identified and an alignment gap (indel) can be classified as an insertion or deletion. In this example, a gap in the orthologous sequence in both the mouse and pig genomes reveals that novel sequence has been inserted in the human genome – as shown by the blue triangle – and that there has been a birth of sequence on the human lineage. Conversely, if there is no gap in the orthologous pig sequence, then a sequence death (pink triangle) is inferred to have taken place on the mouse lineage.

The insertion or deletion of sequence along one of the lineages that separates two species (collectively known as ‘indels’) results in gaps in the sequence alignments which describe the relationship between orthologous sequences within the two genomes being compared. Insertions can be discriminated from deletions by comparing the sequences of three or more genomes simultaneously. The principle of parsimony makes the assumption that the most likely evolutionary history for a set of related sequences is the scenario that can be explained by the minimal number of mutations. In this way, a sequence is defined as having been deleted if it is present in the outgroup species, while an inserted sequence will be absent from this species (Fig. 1). This type of analysis also allows one to identify the lineage along which the insertion or deletion has taken place. Most alignment tools and analysis programs, however, treat these gaps as missing data.

Within the human population, there are likely to be many millions of polymorphic indels which are found in some, but not all, individuals. A study of 79 diverse human genomes reported almost two million small indels 28. However, the limited overlap between this and other studies suggests that this is an under‐estimation of the total number of indels segregating within the human population, and that there are many indels yet to be discovered 28. Longer regions of sequence that has either been inserted or deleted within an individual genome – known as structural variants and generally defined as longer than 1 kb – are less common, and only approximately 20,000 have so far been detected by the 1,000 Genomes Project 6. Within individuals, these variants of different lengths have been found to be associated with differences in gene expression 29, 30.

Polymorphic indels disrupt the coding sequence of over 6% of annotated human genes, but 72% of genes contain an intronic indel 31. The reduced frequency of evolutionarily conserved, functional material within intronic sequences 32 implies that these coding sequence‐disrupting indels are likely to confer a substantial genetic load. Many deletions are shared across populations, and have been present since humans migrated out of Africa 33. The lower average frequency of deletions relative to insertions in the population suggests that sequence loss is more damaging than the birth of new sequence 34.

The distribution of indels throughout the genome has been used to quantify the amount of functional, but lineage‐specific, sequence within the genome. This model assumes that indels occur randomly within the genome and that unexpectedly large distances between indels therefore contain sequence which is preserved by natural selection 35, presumably because this sequence conveys a biological function as yet unknown. By comparing the quantity of sequence defined to be functional using this metric from a range of different pairs of species alignments, the authors determined that this quantity rapidly decreases as the evolutionary distance between the species being compared increased 36. This implies that most functional material is conserved only within a narrow range of related species, and that there must be a rapid turnover of functional sequence and a large quantity of lineage‐specific sequence within mammalian genomes. This rate of sequence turnover is not constant between genomes, and appears to be higher along the mouse lineage, where sequence is preferentially deleted at a particularly high rate 37, 38. The vast majority of this evolutionary volatile sequence is found outside protein‐coding gene borders, and it has been predicted that 110–143 Mb (50%) of functional non‐coding DNA sequence within the human genome has turned over in the last 130 million years 39.

## What mechanisms drive sequence birth and death in the genome?

**Figure 2:**
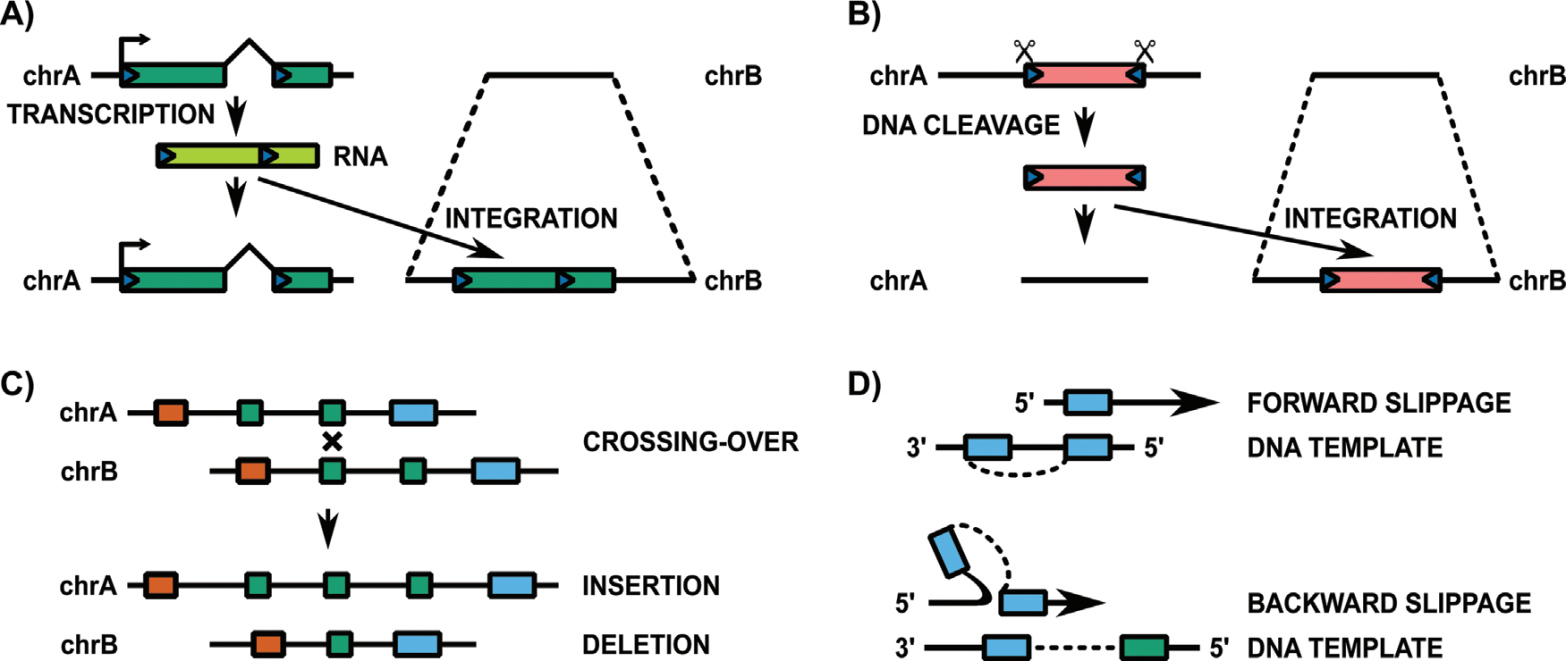
DNA sequences can be inserted and deleted by various mechanisms. Retrotransposons replicate via an RNA intermediate (**A**) while other transposable elements use a cut‐and‐paste mechanism to integrate the DNA sequence at a distant site (**B**). Unequal crossing‐over during cell division (**C**) and replication slippage (**D**) can also result in the birth and death of DNA sequences.

There are a number of molecular mutations that insert or delete sequence in the genome. Transposable elements, which are capable of jumping around the genome, make up approximately half of the human genome 40 and are divided into two major classes. Retrotransposons duplicate via an RNA intermediate (Fig. 2A) before the new copy is re‐integrated into the genome at a distant site. Retrotransposition does not typically include the copying or movement of intronic and surrounding regulatory DNA. The other class of DNA transposons use a cut‐and‐paste mechanism (Fig. 2B) in which the entire DNA sequence is excised and then re‐integrated into the genome. Repetitive elements, and particularly retrotransposons, are enriched at both species‐specific enhancers and gene promoters 41. Promoters, which are the site of RNA polymerase II complex assembly and transcription initiation 42, are enriched for repetitive elements only at sequences that have been inserted, rather than deleted, in both the human and mouse genomes 43. One class of transposons, known as long terminal repeats (LTRs), is particularly common at tissue‐restricted promoters, which is consistent with the previously reported role for LTRs in driving such a limited expression profile 44, 45. Despite the association between simple repetitive elements and sequence deletion 46, no such relationship was found between repetitive sequences and promoters which have been deleted along either the human or mouse lineages 43. Instead, simple repeats were found to be enriched at newly inserted promoters that are broadly expressed, but it remains unknown which types of this family of repeats are responsible or the manner in which they drive widespread expression across tissues.

Sequence can also be inserted or deleted from the genome through the activity of normal cellular processes, such as recombination and replication. Unequal crossing‐over takes place when non‐homologous regions are paired during cell division. This can result in one chromosome gaining sequence and the other losing the same sequence (Fig. 2C), but this exchange of sequence need not be reciprocal as shown here. Indeed, a comparative analysis of the human, chimp and macaque genomes has suggested that recombination is more associated with the gain of sequence than sequence loss in the human genome 47. DNA replication can also create indels through replication slippage (Fig. 2D), if the DNA polymerase skips over a region (known as ‘forward slippage’) to remove sequence or skips back to replicate a region twice (known as ‘backward slippage’), resulting in the insertion of a second copy of the sequence. These replication errors are most frequent at regions containing nearby tandem duplications and, although it has been suggested that they are responsible for most births of recently arisen short insertions in the human lineage 48, replicative errors are actually more likely to be associated with the loss of sequence across the entire genome 47.

Despite reports of sequence gain and loss within the human genome, their accurate discovery remains a difficult task, requiring the development of specialised computational pipelines 49. The two commonly used genome‐wide alignments have been built with different methods – the Ensembl EPO pipeline 50 builds alignments and reconstructs candidate ancestral genome sequences across multiple species simultaneously while the UCSC BLASTZ alignments 51 are generated from small, local sequence alignments of two species which are then extended into larger blocks of related DNA sequences. Multi‐species alignments are then build separately using these pairwise alignments. The difference in these approaches results in substantial differences in the amount of aligning sequence, e.g. UCSC aligns 1.0 Gb (33%) of the human genome to mouse while Ensembl aligns only 820 Mb (26%), and similar discrepancies in the amount of sequence which is estimated to have been gained or lost within the human genome. Furthermore, progressive alignment algorithms incur a greater penalty when creating insertions rather than deletions 52, which hinders robust discrimination of these separate classes of mutations. Further improvements in our ability to identify regions which are inserted and deleted within whole‐genome alignments and the human population are likely to take account of the different mechanisms, and sometimes complicated, evolutionary histories which generate these events. Our increased knowledge of driving forces between these events should improve our ability to predict and accurately detect when an insertion or deletion has truly taken place, rather than as now solely defining them as positions within the genome where alignment pipelines fail to identify orthologous sequences.

## Tissue‐restricted regulatory elements show frequent functional turnover

Genome sequencing projects have been followed by a second wave of functional genomics studies, as exemplified by the work of the ENCODE consortium 53. Functional genomics combines experimental techniques with advances in DNA sequencing to investigate the functional role of genes and other regulatory DNA sequences throughout the genome (see Box 1).

**Figure 3:**
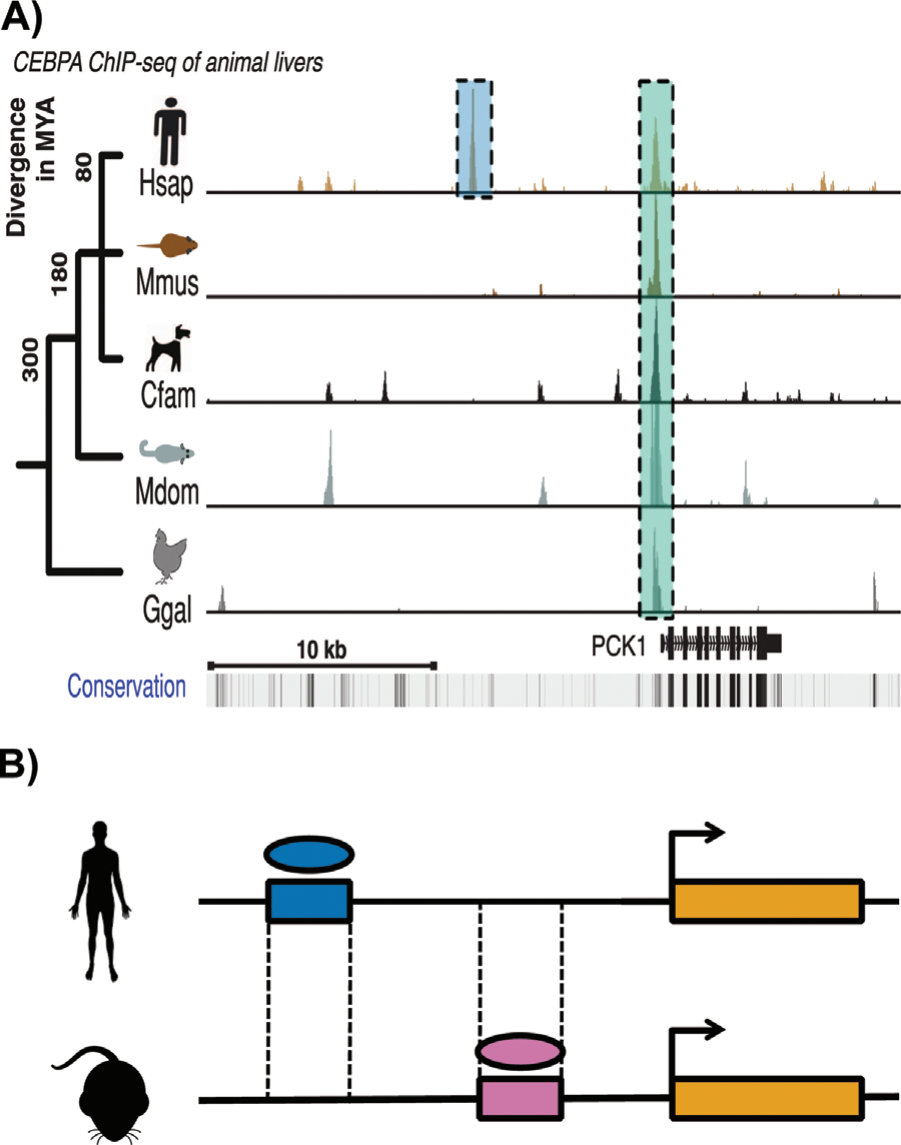
**A:** TF binding at the PCK1 locus in the livers of five vertebrates (human, mouse, dog, monodelphis, chicken). A conserved binding site is highlighted by the green box, and a human‐inserted site by the blue box. Other binding sites show more complicated evolutionary histories. Reproduced with permission from Schmidt et al. 26. **B:** Compensatory turnover of TF binding sites. The locus retains TF binding and a similar transcriptional response in both species, but each TF binding site has turned over.

Large‐scale functional turnover of both transcription factor (TF) binding and promoter locations have been reported. A comparison of four liver‐specific TFs (*FOXA2*, *HNF1A*, *HNF4A* and *HNF6*) in human and mouse revealed that 41–89% of their binding locations within aligning sequence were found in only one of these two species 54, implying a substantial rate of functional turnover. This suggests that there are many births and deaths of these binding sites along the two lineages, but this could not be confirmed from the data published in this study (Fig. 1). This high rate of TF binding turnover in the liver takes place across much of the animal clade (Fig. 3A) 26 and can even be detected between individual rodent lineages, suggesting that these turnover events are evolutionarily very rapid 55.

Complete functional turnover of genetic elements, such as enhancers and promoters, is also frequent between mammalian species. While turnover is less prevalent in *cis*‐regulatory modules that contain multiple TFs bound to the same locus 55, only 279 (less than 1%) of enhancers active in the liver alone are conserved across 10 placental genomes 41. Promoters defined epigenetically by the presence of trimethylated histone H3 lysine 4 (H3K4me3) in the same system seem to be less susceptible than enhancers to this type of functional turnover 41, but this may not reflect the true turnover rate as promoters defined by their transcriptional output using CAGE turn over more frequently 56 (see also below ‘Both sequence and functional turnover contribute to the birth and death of functional promoters’ for a more detailed discussion where promoter turnovers were polarised into births and deaths). There may be further differences between promoter and TF‐binding site functional turnover because these events at promoters are often accompanied by changes to the underlying sequence 43 while binding site locations within rodents can turn over without changes to the underlying DNA sequence 55. It remains unclear what mechanisms, such as the lack of cooperative binding partners or a compaction of the local chromatin state, are responsible for driving these TF turnover events.

The function of DNA‐binding factors or the length of their DNA‐binding motif may also be related to their evolutionary volatility. For example, the binding locations of the insulator protein CTCF – which also has an unusually long binding motif – are much more conserved between mammalian species than most transcription factors, and therefore less likely to be gained or lost between mammalian species 57. Fifteen per cent (5,178/33,966) of alignable binding sites in human are also present in each of macaque, mouse, rat and dog 58. Unlike TFs which often possess tissue‐specific roles in regulating gene expression, CTCF binding sites are largely consistent across tissues 59. CTCF is important for regulating the three‐dimensional structure of the genome, e.g. by insulating transcriptionally active from inactive regions 60 and it also demarcates the borders of DNA sequences which are anchored to the nuclear periphery 61. Like individual CTCF binding sites, this structural role for CTCF appears to be conserved as is the higher‐order genome structure which it regulates 62.

Both the mouse ENCODE 63 and FANTOM5 64 collaborative projects have carried out comparative functional genomics analyses across a range of tissues, and confirmed this rapid functional turnover throughout the human and mouse genomes. Tissue‐restricted elements are more susceptible to turning over, perhaps due to the increased functional constraints on pleiotropic elements that are active across tissues, and the immune system and testis appear to be the tissues throughout the body with the greatest rates of turnover 43. While it is likely that the many of the changes observed within immune cells are driven by positive natural selection to avoid host pathogenicity 65, it is currently unclear to what extent sexual selection and the locally elevated mutation rate at active sites in the germ cells 43, 66, 67, 68 contribute to the functional element turnover within the testis.

## The transcriptional and phenotypic consequences of functional turnover remain unclear

There is evidence that the turnover of an individual binding site can be compensated by the birth of a binding site at a second site within a gene locus (Fig. 3B). For example, approximately 25% of species‐specific TF binding site functional losses in the liver were mirrored by the gain of a separate, species‐specific, binding site within 10 kb 26. Similarly, while 53% of genes targeted in an OCT4 knockdown in embryonic stem cells in both human and mouse contained nearby OCT4‐NANOG binding, only 15% of these binding sites were found at the same position in both species 57. These observations suggest that, while the rapid evolutionary turnover of TF binding sites may be driven by a high mutation rate at these sites 68, this may be matched by a strong selective pressure to prevent a subsequent divergence in the transcriptional output regulated by these factors 69.

Furthermore, the *trans* environment within the cell is more conserved than the individual elements themselves, as TF‐to‐TF interactions 70 and TF network topologies 71 are similar between human and mouse. These results are consistent with the independent observation that human chromosome 21, when inserted into mouse hepatocytes, behaves in largely the same manner as the human chromosome when in human cells. This again suggests that species‐specific binding is due to changes at binding sites themselves but that the same *trans*‐acting TFs can still drive DNA binding in both species 72.

There is little evidence that the majority of these lineage‐specific elements, although defined by their functional activity, are directly involved in transcriptional regulation. It is known that not all TF binding sites have a direct effect on gene expression 73, 74. Binding sites that are identified in the liver and are conserved in multiple mammalian species show greater functional enrichments (e.g. disease ontology annotations), and are found near genes with a higher expression than species‐specific binding sites 75, suggesting that those sites that have been gained or lost down individual lineages are less likely to be functionally important in positively driving gene expression. Furthermore, gene expression and nearby TF binding divergence in the liver do not appear to be generally correlated within closely related mouse species 69. However, these conclusions are contradicted by the observation that mouse‐ and human‐specific binding of the glucocorticoid receptor in macrophages were associated with species‐specific upregulation of neighbouring genes upon glucocorticoid stimulation 76. Further studies of this type are required to determine whether the liver or the macrophage is the more representative system.

Population genetics studies also support the argument that few regulatory elements that have been born along the human lineage possess a biological function. Although enriched for disease‐ and trait‐associated variants, the nucleotide diversity for human‐specific DHSs is relatively high and comparable to that of fourfold‐degenerate sites in exonic sequence, which further suggests that DHSs possess a relatively limited proportion of functional sequence 77. These elements do, however, experience at least some purifying selection, as indicated by their reduced diversity relative to sequences defined biochemically to be inactive 78.

The ultimate test of functionality of lineage‐specific regions is to disrupt them to determine their biological role. Whether, as predicted computationally 36, these elements are frequently responsible for a phenotype has yet to be tested in a systematic manner.

## Functional births and deaths of regulatory elements may be associated with expression changes at nearby genes

With data from only two species, these studies are largely limited to describing turnover events, and matched data from a third species is required to discriminate functional births from deaths along individual lineages (Fig. 1). Shibata et al. 79 measured DNaseI hypersensitive sites (DHSs) in human, chimp and macaque fibroblasts and identified hundreds of gains and losses along both the human and chimp lineages. DHSs that were born along each lineage were associated with up‐regulation of nearby target genes, while DHSs which died were associated with the concomitant down‐regulation of nearby genes. However, most differential gene expression could not be explained by the simple gain or loss of DHS sites. Both gained and lost DHSs were more likely to be experiencing positive selection specifically along the lineage in which they had been gained and lost, respectively. Active enhancers and promoters were similarly identified in embryonic limbs from human, macaque and mouse by profiling the location of acetylated histone H3 lysine 27 (H3K27ac) 80. Promoters identified using this data have gained activity along the human lineage more rapidly than enhancers (13 vs. 11%), but the vast majority of both classes of elements are gained through the co‐option (exaptation) of existing sequence rather than insertion of novel sequence 80. A similar study in the same species mapped two epigenetic marks (dimethylated histone H3 lysine 4, H3K4me2) and H3K27ac during human, macaque and mouse corticogenesis to confirm a high rate of human‐specific promoter and enhancer birth 81. These human‐specific elements were frequently found at, or near to, genes important for cortical development, suggesting that they may play important roles in regulating human‐specific aspects of this important biological process. A collection of histone modifications and protein‐binding sites have also been profiled in matched human, mouse and pig pluripotent stem cells, where divergence in the intensity of these binding factors at gene promoters is correlated with gene expression divergence 82. However, these authors did not explicitly examine functional birth and death of these elements between the lineages studied.

These three‐species experiments also differ from those mentioned above in their methodology for detecting lineage‐specific elements. Those studies described above which focused on liver‐specific transcription factors identified binding regions in each species independently and then defined lineage‐specific regions as those in orthologous regions for which no binding peak had been discovered in other species. The description of an individual region as being lineage‐specific is dependent on the genome‐wide alignments used to identify orthology as these show clear discrepancies in the amount of sequence which can be aligned between species (see ‘What mechanisms drive sequence birth and death in the genome?’ above). The degree of overlap required to identify orthologous regions also affects the detection of functional turnover events. Some studies consider a single 1 bp overlap between regions as sufficient to define them as being conserved while others have required at least a 50% overlap in reciprocal comparisons between species 41, which will reduce the number of lineage‐specific regions that can be identified from the same data. In contrast, these studies describing functional genomics data from other tissues 79, 80, 81, combine these alignments with statistical methods, such as edgeR 83, to detect lineage‐specific regions as those orthologous regions which also show differential levels of histone modifications or chromatin accessibility between species. This approach does not depend on calling peaks in all species and will therefore account for regions with evidence for binding that just misses the threshold for calling a peak as significant within one of the related species. The use of a statistical framework also makes it possible to quantitatively measure the confidence in a single region being truly lineage‐specific and how these regions differ from those identified in the same system which show binding in all species, albeit at significantly different levels. Despite being dependent on replicated functional genomics datasets to make these statistical assessments, these more complex approaches, using more than simple genomic overlaps, will likely be considered the more robust approach to detect functional turnover in future.

## Both sequence and functional turnover contribute to the birth and death of functional promoters

While both mechanisms of birth and death in the genome – sequence and functional turnover – are clearly important contributors to lineage‐specific genomics, it is only recently that they have been explicitly investigated simultaneously in the same experimental system.

The FANTOM5 project, which identified promoter locations across a range of matched human and mouse cell lines and tissues, described the half‐life of promoters when aligned to increasingly divergent species 64. Evolutionary history varied with both expression profile and promoter class, where broadly expressed protein‐coding promoters and tissue‐restricted ncRNA promoters were more deeply conserved. These patterns have been similarly observed within aligning exonic sequence in both protein‐coding genes in human 84 and lncRNAs in *Drosophila* 18.

The sequences of a large number of promoters have been born or died along the human lineage (conservatively 2,472 and 2,818, respectively), since its divergence with mouse 43. As seen for regulatory elements within the ENCODE datasets, the gain and loss of promoters is enriched within immune cells and the testes and brain‐biased promoters were less likely to show either type of sequence turnover. Genes that experienced at least one of these turnover events were enriched for evidence of positive selection acting on their coding sequence, suggesting promoter turnover may be related to adaptive evolution throughout the encoded protein, and not just at the turnover site 79. However, within the human population, both inserted and deleted promoters showed no evidence of either positive or purifying selection, suggesting that, as for the species‐specific TF binding sites described above, many of these may not be phenotypically relevant.

Many promoters whose sequence has been conserved between human and mouse have experienced functional turnover (22 and 13% of aligned promoters in human and mouse, respectively), as they show no detectable evidence of transcription in the opposing species. These species‐specific promoters are specifically associated with decreased evolutionary constraint at the promoter elements 43. Similar levels of evolutionary constraint were seen at promoters with matched, divergent or reduced expression between species, suggesting that differences in transcriptional output were not driven by sequence changes at the promoter or at *cis*‐regulatory elements found at a constant distance from the promoter. This contrasts with the inverse correlation seen between expression and substitution rate divergence at promoters activated in lipopolysaccharide‐stimulated macrophages 85. Whether these differences are specific to the macrophage timecourse profiled here, or are a general feature of stress‐response genes remains unclear.

**Figure 4:**
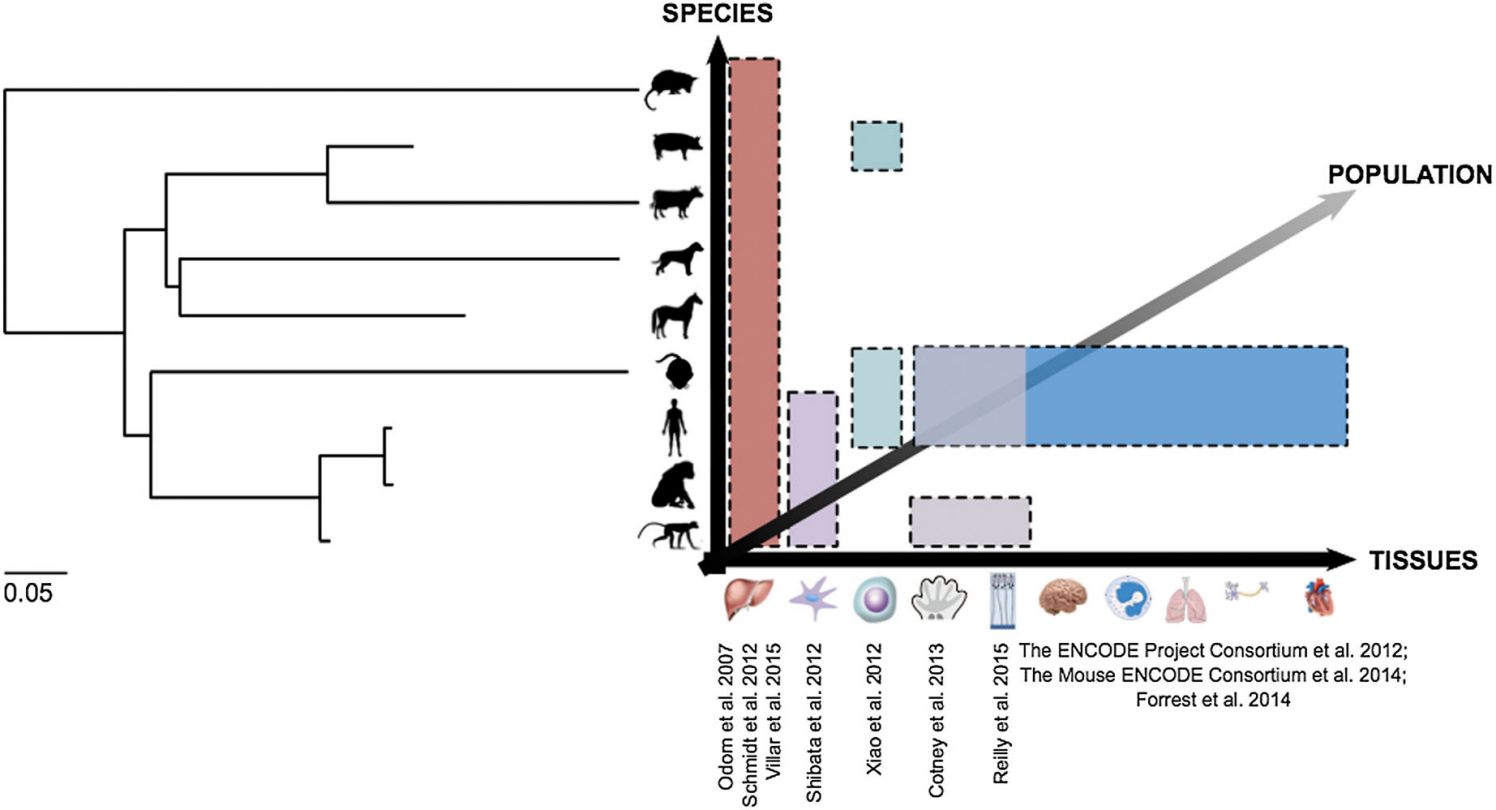
Current state of published, functional genomics data from various mammalian species which are related as shown in the phylogenetic tree. Branch lengths indicate the genome‐wide estimate of the neutral substitution rate at fourfold degenerate sites. ChIP‐seq datasets describing the location of several TFs 53, CTCF 57, H3K27ac and H3K4me3 40 in the liver are available for up to 20 mammalian species, nine of which are shown here. Further studies of three species simultaneously have examined DHSs in fibroblasts 72; various histone modifications and TFs in pluripotent stem cells 75; H3K27ac in the developing limb 73; and H3K4me2 and H3K27ac during corticogenesis 74. The ENCODE, mouse ENCODE and FANTOM consortiums have published large collections of datasets from human and mouse tissues and cell lines. Comparative functional genomics studies within populations of the same species are likely to be a focus of future research.

While sequence gain and loss are clearly important factors in promoter evolution along the mouse and human lineages, the lack of well‐matched data across more species remains the limiting factor for resolving the large number of functional turnovers into births and deaths at aligned sequence. As shown in Fig. 4, beyond the liver, the number of mammalian genomes that has been sequenced outnumbers the number of tissues that have been comprehensively profiled experimentally in multiple species. Even when datasets from multiple tissues are available from consortia such as ENCODE and FANTOM, matched samples are usually only available for human and mouse, hence precluding this discrimination of functional gain from loss at aligning sequences.

## Conclusions and prospects

The birth and death of both sequence and function is a common occurrence within the human genome, and represents an important contributor to genetic diversity. These turnover events have been observed at both distal regulatory elements and functional promoters and confirm that, while a useful predictor, evolutionary conservation is not required to identify functioning, lineage‐specific elements in the human genome.

The availability of large amounts of functional genomics data in both human and mouse have already allowed evolutionary turnover to be investigated across a number of tissues. Extending the available datasets to more distantly related species will permit functional turnover events to be resolved into births and deaths along individual lineages (Fig. 4). Investigating the dynamics of births and deaths within the human population should reveal any phenotypic consequences, and whether they are associated with disease, such as autoimmune disorders, which could aid in the development of personalised strategies to treat these.

Investigations into the combined effects of sequence and functional turnover in the birth and death of genetic elements have only recently been attempted. Further work will likely focus on the direct relationship between these, for example one might ask if functional deaths result in biologically unimportant sequence that is then a target for sequence deletion and the complete removal of the element from the genome.

Despite this work, the biological relevance of these evolutionarily volatile elements still remains unclear. Do they drive the diversification of gene expression profiles, or do they simply represent the neutral churn of redundant genetic elements in the genome? By carefully matching current datasets to increasing amounts of functional genomics data from multiple species 86, we now have an exciting opportunity to reveal the role of evolutionary birth and death in shaping the mammalian genome and its regulatory apparatus.

The author has declared no conflicts of interest.

## References

[1] International Human Genome Sequencing C, Adekoya E , Ait‐Zahra M , Allen N , et al. 2001 Initial sequencing and analysis of the human genome. Nature 409: 860–921. 11237011 10.1038/35057062

[2] Mouse Genome Sequencing C, Waterston RH , Lindblad‐Toh K , Birney E , et al. 2002 Initial sequencing and comparative analysis of the mouse genome. Nature 420: 520–62. 12466850 10.1038/nature01262

[3] Mardis ER. 2013 Next‐generation sequencing platforms. Ann Rev Anal Chem 6: 287–303. 10.1146/annurev-anchem-062012-092628 23560931

[4] Flicek P , Amode MR , Barrell D , Beal K , et al. 2014 Ensembl 2014. Nucleic Acids Res 42: D749–55. 24316576 10.1093/nar/gkt1196 PMC3964975

[5] Levy S , Sutton G , Ng PC , Feuk L , et al. 2007 The diploid genome sequence of an individual human. PLoS Biol 5: e254. 17803354 10.1371/journal.pbio.0050254 PMC1964779

[6] McVean GA , Abecasis GR , Auton A , Brooks LD , et al. 2012 An integrated map of genetic variation from 1,092 human genomes. Nature 491: 56–65. 23128226 10.1038/nature11632 PMC3498066

[7] Gudbjartsson DF , Helgason H , Gudjonsson SA , Zink F , et al. 2015 Large‐scale whole‐genome sequencing of the Icelandic population. Nat Genet 47: 435–44. 25807286 10.1038/ng.3247

[8] Douzery EJ , Snell EA , Bapteste E , Delsuc F , et al. 2004 The timing of eukaryotic evolution: does a relaxed molecular clock reconcile proteins and fossils? Proc Natl Acad Sci U S A 101: 15386–91. 15494441 10.1073/pnas.0403984101 PMC524432

[9] Hahn MW , Wray GA. 2002 The g‐value paradox. Evol Dev 4: 73–5. 12004964 10.1046/j.1525-142x.2002.01069.x

[10] Li CY , Zhang Y , Wang Z , Zhang Y , et al. 2010 A human‐specific de novo protein‐coding gene associated with human brain functions. PLoS Comput Biol 6: e1000734. 20376170 10.1371/journal.pcbi.1000734 PMC2845654

[11] Kaessmann H. 2010 Origins, evolution, and phenotypic impact of new genes. Genome Res 20: 1313–26. 20651121 10.1101/gr.101386.109 PMC2945180

[12] Church DM , Goodstadt L , Hillier LW , Zody MC , et al. 2009 Lineage‐specific biology revealed by a finished genome assembly of the mouse. PLoS Biol 7: e1000112‐e. 19468303 10.1371/journal.pbio.1000112 PMC2680341

[13] Bartel DP. 2009 MicroRNAs: target recognition and regulatory functions. Cell 136: 215–33. 19167326 10.1016/j.cell.2009.01.002 PMC3794896

[14] Wheeler BM , Heimberg AM , Moy VN , Sperling EA , et al. 2009 The deep evolution of metazoan microRNAs. Evol Dev 11: 50–68. 19196333 10.1111/j.1525-142X.2008.00302.x

[15] Ponting CP , Oliver PL , Reik W. 2009 Evolution and functions of long noncoding RNAs. Cell 136: 629–41. 19239885 10.1016/j.cell.2009.02.006

[16] Ponjavic J , Ponting CP , Lunter G. 2007 Functionality or transcriptional noise? Evidence for selection within long noncoding RNAs. Genome Res 17: 556–65. 17387145 10.1101/gr.6036807 PMC1855172

[17] Marques AC , Ponting CP. 2009 Catalogues of mammalian long noncoding RNAs: modest conservation and incompleteness. Genome Biol 10: R124‐R. 19895688 10.1186/gb-2009-10-11-r124 PMC3091318

[18] Young RS , Marques AC , Tibbit C , Haerty W , et al. 2012 Identification and properties of 1,119 candidate lincRNA loci in the Drosophila melanogaster genome. Genome Biol Evol 4: 427–42. 22403033 10.1093/gbe/evs020 PMC3342871

[19] Bulger M , Groudine M. 2010 Enhancers: the abundance and function of regulatory sequences beyond promoters. Dev Biol 339: 250–7. 20025863 10.1016/j.ydbio.2009.11.035 PMC3060611

[20] Pennacchio LA , Ahituv N , Moses AM , Prabhakar S , et al. 2006 In vivo enhancer analysis of human conserved non‐coding sequences. Nature 444: 499–502. 17086198 10.1038/nature05295

[21] Heinen TJ , Staubach F , Haming D , Tautz D. 2009 Emergence of a new gene from an intergenic region. Curr Biol 19: 1527–31. 19733073 10.1016/j.cub.2009.07.049

[22] Hu HY , He L , Fominykh K , Yan Z , et al. 2012 Evolution of the human‐specific microRNA miR‐941. Nat Commun 3: 1145. 23093182 10.1038/ncomms2146 PMC3493648

[23] Prabhakar S , Visel A , Akiyama JA , Shoukry M , et al. 2008 Human‐specific gain of function in a developmental enhancer. Science 321: 1346–50. 18772437 10.1126/science.1159974 PMC2658639

[24] McLean CY , Reno PL , Pollen AA , Bassan AI , et al. 2011 Human‐specific loss of regulatory DNA and the evolution of human‐specific traits. Nature 471: 216–9. 21390129 10.1038/nature09774 PMC3071156

[25] Kutter C , Watt S , Stefflova K , Wilson MD , et al. 2012 Rapid turnover of long noncoding RNAs and the evolution of gene expression. PLoS Genet 8: e1002841. 22844254 10.1371/journal.pgen.1002841 PMC3406015

[26] Schmidt D , Wilson MD , Ballester B , Schwalie PC , et al. 2010 Five‐vertebrate ChIP‐seq reveals the evolutionary dynamics of transcription factor binding. Science 328: 1036–40. 20378774 10.1126/science.1186176 PMC3008766

[27] Wittkopp PJ , Kalay G. 2012 Cis‐regulatory elements: molecular mechanisms and evolutionary processes underlying divergence. Nat Rev Genet 13: 59–69. 22143240 10.1038/nrg3095

[28] Mills RE , Pittard WS , Mullaney JM , Farooq U , et al. 2011 Natural genetic variation caused by small insertions and deletions in the human genome. Genome Res 21: 830–9. 21460062 10.1101/gr.115907.110 PMC3106316

[29] Stranger BE , Forrest MS , Dunning M , Ingle CE , et al. 2007 Relative impact of nucleotide and copy number variation on gene expression phenotypes. Science 315: 848–53. 17289997 10.1126/science.1136678 PMC2665772

[30] Gymrek M , Willems T , Guilmatre A , Zeng H , et al. 2016 Abundant contribution of short tandem repeats to gene expression variation in humans. Nat Genet 48: 22–9. 26642241 10.1038/ng.3461 PMC4909355

[31] Chen FC , Chen CJ , Li WH , Chuang TJ. 2007 Human‐specific insertions and deletions inferred from mammalian genome sequences. Genome Res 17: 16–22. 17095709 10.1101/gr.5429606 PMC1716262

[32] Siepel A , Bejerano G , Pedersen JS , Hinrichs AS , et al. 2005 Evolutionarily conserved elements in vertebrate, insect, worm, and yeast genomes. Genome Res 15: 1034–50. 16024819 10.1101/gr.3715005 PMC1182216

[33] McCarroll SA , Hadnott TN , Perry GH , Sabeti PC , et al. 2006 Common deletion polymorphisms in the human genome. Nat Genet 38: 86–92. 16468122 10.1038/ng1696

[34] Sjodin P , Bataillon T , Schierup MH. 2010 Insertion and deletion processes in recent human history. PLoS ONE 5: e8650. 20098729 10.1371/journal.pone.0008650 PMC2808225

[35] Lunter G , Ponting CP , Hein J. 2006 Genome‐wide identification of human functional DNA using a neutral indel model. PLoS Comput Biol 2: e5. 16410828 10.1371/journal.pcbi.0020005 PMC1326222

[36] Meader S , Ponting CP , Lunter G. 2010 Massive turnover of functional sequence in human and other mammalian genomes. Genome Res 20: 1335–43. 20693480 10.1101/gr.108795.110 PMC2945182

[37] Laurie S , Toll‐Riera M , Rado‐Trilla N , Alba MM. 2012 Sequence shortening in the rodent ancestor. Genome Res 22: 478–85. 22128134 10.1101/gr.121897.111 PMC3290783

[38] Taylor MS , Ponting CP , Copley RR. 2004 Occurrence and consequences of coding sequence insertions and deletions in Mammalian genomes. Genome Res 14: 555–66. 15059996 10.1101/gr.1977804 PMC383299

[39] Rands CM , Meader S , Ponting CP , Lunter G. 2014 8.2% of the human genome is constrained: variation in rates of turnover across functional element classes in the human lineage. PLoS Genet 10: e1004525. 25057982 10.1371/journal.pgen.1004525 PMC4109858

[40] Kuhn RM , Karolchik D , Zweig AS , Trumbower H , et al. 2007 The UCSC genome browser database: update 2007. Nucleic Acids Res 35: D668–73. 17142222 10.1093/nar/gkl928 PMC1669757

[41] Villar D , Berthelot C , Aldridge S , Rayner TF , et al. 2015 Enhancer evolution across 20 mammalian species. Cell 160: 554–66. 25635462 10.1016/j.cell.2015.01.006 PMC4313353

[42] Sandelin A , Carninci P , Lenhard B , Ponjavic J , et al. 2007 Mammalian RNA polymerase II core promoters: insights from genome‐wide studies. Nat Rev Genet 8: 424–36. 17486122 10.1038/nrg2026

[43] Young RS , Hayashizaki Y , Andersson R , Sandelin A , et al. 2015 The frequent evolutionary birth and death of functional promoters in mouse and human. Genome Res 25: 1546–57. 26228054 10.1101/gr.190546.115 PMC4579340

[44] Faulkner GJ , Kimura Y , Daub CO , Wani S , et al. 2009 The regulated retrotransposon transcriptome of mammalian cells. Nat Genet 41: 563–71. 19377475 10.1038/ng.368

[45] Fort A , Hashimoto K , Yamada D , Salimullah M , et al. 2014 Deep transcriptome profiling of mammalian stem cells supports a regulatory role for retrotransposons in pluripotency maintenance. Nat Genet 46: 558–66. 24777452 10.1038/ng.2965

[46] Usdin K. 2008 The biological effects of simple tandem repeats: lessons from the repeat expansion diseases. Genome Res 18: 1011–9. 18593815 10.1101/gr.070409.107 PMC3960014

[47] Kvikstad EM , Tyekucheva S , Chiaromonte F , Makova KD. 2007 A macaque's‐eye view of human insertions and deletions: differences in mechanisms. PLoS Comput Biol 3: 1772–82. 17941704 10.1371/journal.pcbi.0030176 PMC1976337

[48] Messer PW , Arndt PF. 2007 The majority of recent short DNA insertions in the human genome are tandem duplications. Mol Biol Evol 24: 1190–7. 17322553 10.1093/molbev/msm035

[49] Mills RE , Luttig CT , Larkins CE , Beauchamp A , et al. 2006 An initial map of insertion and deletion (INDEL) variation in the human genome. Genome Res 16: 1182–90. 16902084 10.1101/gr.4565806 PMC1557762

[50] Paten B , Herrero J , Beal K , Fitzgerald S , et al. 2008 Enredo and Pecan: genome‐wide mammalian consistency‐based multiple alignment with paralogs. Genome Res 18: 1814–28. 18849524 10.1101/gr.076554.108 PMC2577869

[51] Schwartz S , Kent WJ , Smit A , Zhang Z , et al. 2003 Human‐mouse alignments with BLASTZ. Genome Res 13: 103–7. 12529312 10.1101/gr.809403 PMC430961

[52] Loytynoja A , Goldman N. 2008 Phylogeny‐aware gap placement prevents errors in sequence alignment and evolutionary analysis. Science 320: 1632–5. 18566285 10.1126/science.1158395

[53] Consortium EP. 2012 An integrated encyclopedia of DNA elements in the human genome. Nature 489: 57–74. 22955616 10.1038/nature11247 PMC3439153

[54] Odom DT , Dowell RD , Jacobsen ES , Gordon W , et al. 2007 Tissue‐specific transcriptional regulation has diverged significantly between human and mouse. Nat Genet 39: 730–2. 17529977 10.1038/ng2047 PMC3797512

[55] Stefflova K , Thybert D , Wilson MD , Streeter I , et al. 2013 Cooperativity and rapid evolution of cobound transcription factors in closely related mammals. Cell 154: 530–40. 23911320 10.1016/j.cell.2013.07.007 PMC3732390

[56] Frith MC , Ponjavic J , Fredman D , Kai C , et al. 2006 Evolutionary turnover of mammalian transcription start sites. Genome Res 16: 713–22. 16687732 10.1101/gr.5031006 PMC1473182

[57] Kunarso G , Chia NY , Jeyakani J , Hwang C , et al. 2010 Transposable elements have rewired the core regulatory network of human embryonic stem cells. Nat Genet 42: 631–4. 20526341 10.1038/ng.600

[58] Schmidt D , Schwalie PC , Wilson MD , Ballester B , et al. 2012 Waves of retrotransposon expansion remodel genome organization and CTCF binding in multiple mammalian lineages. Cell 148: 335–48. 22244452 10.1016/j.cell.2011.11.058 PMC3368268

[59] Shen Y , Yue F , McCleary DF , Ye Z , et al. 2012 A map of the cis‐regulatory sequences in the mouse genome. Nature 488: 116–20. 22763441 10.1038/nature11243 PMC4041622

[60] Herold M , Bartkuhn M , Renkawitz R. 2012 CTCF: insights into insulator function during development. Development 139: 1045–57. 22354838 10.1242/dev.065268

[61] Guelen L , Pagie L , Brasset E , Meuleman W , et al. 2008 Domain organization of human chromosomes revealed by mapping of nuclear lamina interactions. Nature 453: 948–51. 18463634 10.1038/nature06947

[62] Chambers EV , Bickmore WA , Semple CA. 2013 Divergence of mammalian higher order chromatin structure is associated with developmental loci. PLoS Comput Biol 9: e1003017. 23592965 10.1371/journal.pcbi.1003017 PMC3617018

[63] Yue F , Cheng Y , Breschi A , Vierstra J , et al. 2014 A comparative encyclopedia of DNA elements in the mouse genome. Nature 515: 355–64. 25409824 10.1038/nature13992 PMC4266106

[64] Forrest AR , Kawaji H , Rehli M , Baillie JK , et al. 2014 A promoter‐level mammalian expression atlas. Nature 507: 462–70. 24670764 10.1038/nature13182 PMC4529748

[65] Vallender EJ , Lahn BT. 2004 Positive selection on the human genome. Hum Mol Genet 13: R245–54. 15358731 10.1093/hmg/ddh253

[66] Haygood R , Fedrigo O , Hanson B , Yokoyama KD , et al. 2007 Promoter regions of many neural‐ and nutrition‐related genes have experienced positive selection during human evolution. Nat Genet 39: 1140–4. 17694055 10.1038/ng2104

[67] Taylor MS , Massingham T , Hayashizaki Y , Carninci P , et al. 2008 Rapidly evolving human promoter regions. Nat Genet 40: 1262–3; author reply 3–4. 18957975 10.1038/ng1108-1262

[68] Reijns MA , Kemp H , Ding J , de Proce SM , et al. 2015 Lagging‐strand replication shapes the mutational landscape of the genome. Nature 518: 502–6. 25624100 10.1038/nature14183 PMC4374164

[69] Wong ES , Thybert D , Schmitt BM , Stefflova K , et al. 2015 Decoupling of evolutionary changes in transcription factor binding and gene expression in mammals. Genome Res 25: 167–78. 25394363 10.1101/gr.177840.114 PMC4315291

[70] Neph S , Vierstra J , Stergachis AB , Reynolds AP , et al. 2012 An expansive human regulatory lexicon encoded in transcription factor footprints. Nature 489: 83–90. 22955618 10.1038/nature11212 PMC3736582

[71] Stergachis AB , Neph S , Sandstrom R , Haugen E , et al. 2014 Conservation of trans‐acting circuitry during mammalian regulatory evolution. Nature 515: 365–70. 25409825 10.1038/nature13972 PMC4405208

[72] Wilson MD , Barbosa‐Morais NL , Schmidt D , Conboy CM , et al. 2008 Species‐specific transcription in mice carrying human chromosome 21. Science 322: 434–8. 18787134 10.1126/science.1160930 PMC3717767

[73] Biggin MD. 2011 Animal transcription networks as highly connected, quantitative continua. Dev Cell 21: 611–26. 22014521 10.1016/j.devcel.2011.09.008

[74] Bradley RK , Li XY , Trapnell C , Davidson S , et al. 2010 Binding site turnover produces pervasive quantitative changes in transcription factor binding between closely related Drosophila species. PLoS Biol 8: e1000343. 20351773 10.1371/journal.pbio.1000343 PMC2843597

[75] Ballester B , Medina‐Rivera A , Schmidt D , Gonzalez‐Porta M , et al. 2014 Multi‐species, multi‐transcription factor binding highlights conserved control of tissue‐specific biological pathways. eLife 3: e02626. 25279814 10.7554/eLife.02626 PMC4359374

[76] Jubb AW , Young RS , Hume DA , Bickmore WA. 2016 Enhancer turnover is associated with a divergent transcriptional response to glucocorticoid in mouse and human macrophages. J Immunol 196: 813–22. 26663721 10.4049/jimmunol.1502009 PMC4707550

[77] Vierstra J , Rynes E , Sandstrom R , Zhang M , et al. 2014 Mouse regulatory DNA landscapes reveal global principles of cis‐regulatory evolution. Science 346: 1007–12. 25411453 10.1126/science.1246426 PMC4337786

[78] Ward LD , Kellis M. 2012 Evidence of abundant purifying selection in humans for recently acquired regulatory functions. Science 337: 1675–8. 22956687 10.1126/science.1225057 PMC4104271

[79] Shibata Y , Sheffield NC , Fedrigo O , Babbitt CC , et al. 2012 Extensive evolutionary changes in regulatory element activity during human origins are associated with altered gene expression and positive selection. PLoS Genet 8: e1002789. 22761590 10.1371/journal.pgen.1002789 PMC3386175

[80] Cotney J , Leng J , Yin J , Reilly SK , et al. 2013 The evolution of lineage‐specific regulatory activities in the human embryonic limb. Cell 154: 185–96. 23827682 10.1016/j.cell.2013.05.056 PMC3785101

[81] Reilly SK , Yin J , Ayoub AE , Emera D , et al. 2015 Evolutionary genomics. Evolutionary changes in promoter and enhancer activity during human corticogenesis. Science 347: 1155–9. 25745175 10.1126/science.1260943 PMC4426903

[82] Xiao S , Xie D , Cao X , Yu P , et al. 2012 Comparative epigenomic annotation of regulatory DNA. Cell 149: 1381–92. 22682255 10.1016/j.cell.2012.04.029 PMC3372872

[83] Robinson MD , McCarthy DJ , Smyth GK. 2010 edgeR: a bioconductor package for differential expression analysis of digital gene expression data. Bioinformatics 26: 139–40. 19910308 10.1093/bioinformatics/btp616 PMC2796818

[84] Winter EE , Goodstadt L , Ponting CP. 2004 Elevated rates of protein secretion, evolution, and disease among tissue‐specific genes. Genome Res 14: 54–61. 14707169 10.1101/gr.1924004 PMC314278

[85] Schroder K , Irvine KM , Taylor MS , Bokil NJ , et al. 2012 Conservation and divergence in toll‐like receptor 4‐regulated gene expression in primary human versus mouse macrophages. Proc Natl Acad Sci U S A 109: E944–53. 22451944 10.1073/pnas.1110156109 PMC3341041

[86] Andersson L , Archibald AL , Bottema CD , Brauning R , et al. 2015 Coordinated international action to accelerate genome‐to‐phenome with FAANG, the Functional Annotation of Animal Genomes project. Genome Biol 16: 57. 25854118 10.1186/s13059-015-0622-4 PMC4373242

